# *Tropheryma whipplei* colonization in HIV-infected individuals is not associated with lung function or inflammation

**DOI:** 10.1371/journal.pone.0205065

**Published:** 2018-10-04

**Authors:** Shulin Qin, Emily Clausen, Seyed Mehdi Nouraie, Lawrence Kingsley, Deborah McMahon, Eric Kleerup, Laurence Huang, Elodie Ghedin, Ruth M. Greenblatt, Alison Morris

**Affiliations:** 1 Departments of Medicine, University of Pittsburgh, Pittsburgh, Pennsylvania, United States of America; 2 Department of Infectious Diseases and Microbiology, Graduate School of Public Health, University of Pittsburgh, Pittsburgh, Pennsylvania, United States of America; 3 Department of Medicine, University of California Los Angeles, Los Angeles, California, United States of America; 4 Department of Medicine, University of California San Francisco, San Francisco, California, United States of America; 5 Department of Biology, Center for Genomics and Systems Biology, and Global Institute of Public Health, New York University, New York, New York, United States of America; 6 Department of Clinical Pharmacy, University of California, San Francisco, San Francisco, California, United States of America; 7 Departments of Immunology, University of Pittsburgh, Pittsburgh, Pennsylvania, United States of America; Washington University in Saint Louis, UNITED STATES

## Abstract

Studies demonstrate that *Tropheryma whipplei (T*. *whipplei)* is present in the lungs of healthy individuals without acute respiratory symptoms or acute respiratory infection and is more common in the lungs of HIV-infected individuals and in smokers. The impact of *T*. *whipplei* colonization in the lung on local inflammation and pulmonary dysfunction in HIV-infected individuals is currently unknown. In this study, we performed specific polymerase chain reaction (PCR) and sequencing for *T*. *whipplei* in bronchoalveolar lavage (BAL) and induced sputum (IS) samples in 76 HIV-infected participants from three clinical sites. Pulmonary function and proinflammatory cytokine and chemokine levels in BAL were measured. Frequency of *T*. *whipplei* in either BAL or IS was 43.4%. The sensitivity and specificity of IS compared to BAL for detection of *T*. *whipplei* was 92.3% and 84.2%, respectively, and isolates of *T*. *whipplei* in the BAL and IS in the same subject shared genetic identity. Pulmonary function measures were not associated with *T*. *whipplei* colonization, and proinflammatory cytokine and chemokine levels in BAL and plasma as well as percentages of inflammatory cells in BAL and IS were not higher in colonized individuals. Overall, these results indicate that *T*. *whipplei* colonization in the lung is common, but may not be associated with decreased pulmonary function or inflammation in HIV-infected individuals.

## Introduction

The organism *Tropheryma whipplei (T*. *whipplei)* causes Whipple’s disease, an infectious disease that primarily involves the gastrointestinal tract [1]. The prevalence of pulmonary involvement in Whipple’s disease is about 13 percent, and its clinical respiratory features include shortness of breath, dry cough and chest pain[2]. Recent microbiome studies demonstrate that *T*. *whipplei* is present in the lungs of healthy individuals without acute respiratory symptoms or acute respiratory infection and more common in the lungs of HIV-infected individuals and in smokers with antiretroviral therapy (ART) significantly reducing its relative abundance [3–5].

Chronic obstructive pulmonary disease (COPD) is a common complication in HIV-infected individuals, as 7–21% of HIV-infected individuals have obstructive ventilatory defects and 50–64% have reduced diffusing capacity for carbon monoxide (DLco) [6–10]. Despite its frequency in HIV, understanding of the pathogenesis of COPD in HIV is incomplete. Immune dysfunction caused by HIV infection could lead to microbial changes in the lung, and alterations of the lung microbiome could be involved in the development and progression of COPD [11,12]. It has been reported that low levels of organisms such as *Pneumocystis jirovecii* (*P*. *jirovecii*) are associated with COPD in HIV-infected individuals [13,14], but associations between *T*. *whipplei* colonization in the lung, local and systemic inflammation, and pulmonary dysfunction in HIV-infected individuals are unknown. If *T*. *whipplei* contributes to inflammation and lung damage in HIV, it might be a reversible contributor to COPD as it can be treated with antibiotics. We therefore investigated the detection of *T*. *whipplei* by bronchoscopy and induced sputum and the relationship to lung and systemic inflammation and pulmonary function in HIV-infected individuals.

## Methods

### Participants and sample collection

We included 76 HIV-infected participants who had undergone bronchoscopy with bronchoalveolar lavage (BAL) as part of the Pittsburgh, San Francisco and Los Angeles sites of the Lung HIV Microbiome Project (LHMP) cohort (https://lhmp.bsc.gwu.edu/) or the Pittsburgh Lung HIV Study cohort and had sufficient BAL samples available for analysis [15–17]. Inclusion criteria in the LHMP were (1) age 18 to 75 years old; (2) HIV-infected. Exclusion criteria were (1) an upper or lower respiratory tract infection within the past month, acute onset of shortness of breath, cough, fever; or use of antibiotics within the past six months; (2) Heart conditions such as tachycardia, angina, or arrhythmias; (3) Significant or uncontrolled systemic diseases. The study protocol was approved by the Institutional Review Boards at the University of Pittsburgh, the University of California San Francisco, and University of California Los Angeles. Informed consent was obtained from each participant.

Demographic and clinical data were collected by standardized participant interview including age, gender, smoking history, current antiretroviral therapy (ART), and history of prior pneumonia. CD4 cell count, and HIV RNA level (viral load) were confirmed by chart review or direct testing. Bronchoscopy was performed according to a standardized protocol developed to minimize oral contamination [3,18]. Bronchoalveolar lavage was performed in a subsegment of the right middle lobe or lingula with up to a maximum of 200 ml of 0.9% sterile saline instilled. Pre- and post-bronchodilator spirometry and DLco were performed per American Thoracic Society/European Respiratory Society guidelines [19,20]. Hankinson and Neas equations were used to determine percent predicted values of spirometry [21,22] and DLco, respectively. DLco was corrected for hemoglobin and carboxyhemoglobin [22]. Induced sputum (IS) samples were obtained from 64 of 76 (84.2%) participants as previously described [23].

### *T*. *whipplei* PCR and sequence analysis

DNA was isolated from 5ml of whole BAL and 1ml of IS using the PowerSoil DNA isolation kit (MoBio, Carlsbad, CA). *T*. *whipplei* was detected by using *T*. *whipplei* hsp65 specific nested-PCR [24]. Negative and positive *T*. *whipplei* controls were included in all reactions. Amplified products were purified using Agencourt AMPure XP PCR Purification kit (Beckman Coulter, Brea, CA), and then sequenced using specific primers [24] by the Genomics and Proteomics Core laboratories at the University of Pittsburgh. To determine the genotype of *T*. *whipplei*, we performed Sanger sequencing of amplified products of the partial *T*. *whipplei* hsp65 gene. CLC Main Workbench 6.5 and the Molecular Evolutionary Genetics Analysis version 6 (MEGA6) software packages [25] were used for analyses of *T*. *whipplei* hsp65 partial gene sequences.

### Measurement of lung and peripheral circulating proinflammatory cytokines and chemokines

BAL supernatant samples were obtained by centrifuging 50ml of BAL fluid at 300g for 7 minutes at 4°C. Levels of proinflammatory cytokines and chemokines in BAL supernatant and plasma were measured using a Bio-Plex human cytokine, chemokine 9-plex assay kit (Bio-Rad, Hercules, California, USA) per the manufacturer’s protocol utilizing recommended standard curve concentrations. The 9-plex assay kit quantified interleukin-1 receptor antagonist (IL-1RA), interleukin (IL)-4, IL-6, IL-13, CCL3, CCL4, CCL5, CCL11 and tumor necrosis factor alpha (TNF-α). Luminex data were analyzed using Bio-Plex Manager software (Bio-Rad). Cytokine and chemokine concentrations in the BAL supernatant were determined after correction of dilution factor as ratio of serum urea/BAL urea [26].

### Cytology of BAL fluid and IS samples

Cytospin slides (25,000 cells per slide) of BAL and IS samples were generated. The slides were stained with Diff Quick and 300–500 cell differential counts were performed to determine the numbers and percentages of inflammatory cells.

### Statistical analysis

Sensitivity of IS compared to BAL for detection of *T*. *whipplei* was determined. Demographic and clinical characteristics including pulmonary function variables, and levels of proinflammatory cytokines and chemokines in BAL supernatant and plasma as well as cytology of BAL fluid and IS were compared between participants with and without detectable *T*. *whipplei* in either BAL or IS using Welch's t-test or Kruskal-Wallis test for continuous variables and Fisher exact test for categorical variables. We used ANOVA to test the gender and smoking adjusted effect of *T*. *whipplei* on pulmonary function tests (as continuous variables). We also used Firth’s penalized logistic regression to test this effect on FEV1/FVC less than 70 percent and DLco less than 80 percent predicted. We had 80% power to detect a difference of 11% or more between *T*. *whipplei* groups for Post FVC%. All analyses were performed in Stata 14.2 (StataCorp LLC, College Station, TX).

## Results

### Frequency and genotype of *T*. *whipplei* in the lung

Thirty-three of 76 HIV-infected participants (43.4%) had *T*. *whipplei* detected in either BAL or IS (Table 1). *T*. *whipplei* was detected in 27 of 76 BAL samples (35.5%) and 30 of 64 IS samples (46.9%) by both specific nested PCR and sequencing techniques. Sixty-four participants had both BAL and IS samples collected; 24 of 26 (92.3%) *T*. *whipplei*-positive BAL samples also had matching *T*. *whipplei*-positive IS samples (Table 2) and one subject with BAL *T*. *whipplei* positive did not have an IS sample. There were 6 *T*. *whipplei*-negative BAL samples that had matching positive IS samples (Table 2). Sensitivity and specificity of IS compared to BAL for detection of *T*. *whipplei* was 92.3% and 84.2%, respectively. We analyzed and compared our previous 16s rRNA and current PCR data on frequency of *T*. *whipplei* in 39 BAL samples from this study and found that *T*. *whipplei* was detected in 33.3% (13/39) and 30.8% (12/39) BAL samples by *T*. *whipplei* PCR and 16s rRNA gene sequencing, respectively, indicating that *T*. *whipplei* hsp65 specific-PCR assay has greater sensitivity than 16S rRNA gene sequencing.

**Table 1:** Demographic and clinical characteristics of participants.

Characteristic	*T*. *whipplei-* (n = 43)	*T*. *whipplei+* (n = 33)	*P*-value*
**Age, median year (IQR)**	53 (46–59)	52 (46–58)	0.87
**Female, sex, n (%)**	8 (19)	16 (48)	0.007
**CD4, median (IQR)**	616 (410–892)	630 (379–876)	0.89
**Viral load, median (IQR) copies per ml,**	109 (27–2478)	102 (31–4965)	0.85
**ART, n (%)**	41 (95)	30 (91)	0.65
**Prior pneumonia, n (%)**	9 (21%)	5 (15%)	0.57
**Smoker, n (%)**	24 (56)	24 (73)	0.16
**Cigarette pack year, median (IQR)**	3.2 (0–13.5)	11.9 (0–21.8)	0.23
**Post-FEV1/FVC<0.7, n (%)**	6 (15)	6 (19)	0.75
**DLco<0.8, n (%)**	26 (67)	18 (58)	0.62

IQR, interquartile range; ART, antiretroviral therapy.

* *p* values were calculated using t-test test for continuous variables and Fisher exact test for categorical.

**Table 2:** Frequency of *T*. *whipplei* in bronchoalveolar lavage and induced sputum from 64 participants with both bronchoalveolar lavage and induced sputum available.

*T*. *whipplei* (Bronchoalveolar lavage)	*T*. *whipplei* (Induced sputum) Positive	*T*. *whipplei* (Induced sputum) Negative	Total
**Positive**	24 (92.3%)	2	26
**Negative**	6	32 (84.2%)	38
**Total**	30	34	64

BAL, Bronchoalveolar lavage, IS, Induced sputum

**Fig 1:**
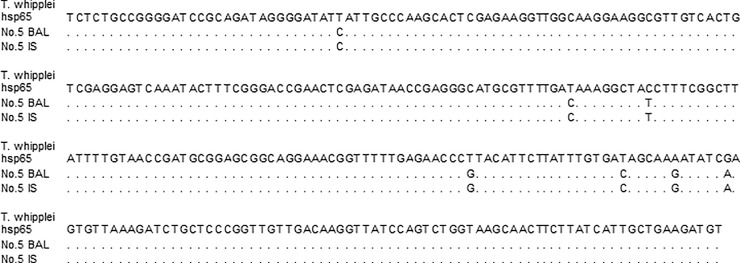
Partial *hsp65* gene sequence of *T*. *whipplei* in bronchoalveolar lavage and induced sputum samples from the same participant. The gene sequences of the reference *T*. *whipplei* hsp65 and the isolates of *T*. *whipplei* from number 5 participant’s BAL and IS were analyzed using Molecular Evolutionary Genetics Analysis version 6 (MEGA6) software packages. Both isolates of *T*. *whipplei* in the BAL and IS in the same participant had mutations in the hsp65 gene and shared genetic identity. BAL, Bronchoalveolar lavage; IS, induced sputum; hsp65, heat-shock protein 65 gene.

The majority of *T*. *whipplei* detected in BAL (63.0%, 17/27) or IS (66.7%, 20/30) were identical to the reference sequence (wild-type). *T*. *whipplei* containing one or more mutations in the hsp65 gene was found in 37.0% of BAL samples (10/27) and in 33.3% of IS (10/30), respectively. In addition, we compared the genotype of *T*. *whipplei* in 24 participants who had *T*. *whipplei* in both BAL and IS and found 100% concordance (Table 3). Fifteen participants had wild-type *T*. *whipplei*, and nine had the same mutations in the hsp65 gene in both BAL and IS (Table 3 and Fig 1).

**Table 3:** Comparison of partial *hsp65* gene sequence of *T*. *whipplei* in bronchoalveolar lavage and induced sputum samples.

**Subject**	**Bronchoalveolar lavage**	**Induced sputum**
**1**	Wild-type	Wild-type
**2**	Wild-type	Wild-type
**3**	Mutation (1nt)	Mutation (1nt)
**4**	Wild-type	Wild-type
**5**	Mutation (7nt)	Mutation (7nt)
**6**	Wild-type	Wild-type
**7**	Wild-type	Wild-type
**8**	Wild-type	Wild-type
**9**	Wild-type	Wild-type
**10**	Mutation (6nt)	Mutation (6nt)
**11**	Mutation (7nt)	Mutation (7nt)
**12**	Wild-type	Wild-type
**13**	Mutation (4nt)	Mutation (4nt)
**14**	Mutation (9nt)	Mutation (9nt)
**15**	Wild-type	Wild-type
**16**	Mutation (4nt)	Mutation (4nt)
**17**	Wild-type	Wild-type
**18**	Wild-type	Wild-type
**19**	Wild-type	Wild-type
**20**	Mutation (6nt)	Mutation (6nt)
**21**	Wild-type	Wild-type
**22**	Wild-type	Wild-type
**23**	Mutation (6nt)	Mutation (6nt)
**24**	Wild-type	Wild-type

BAL, Bronchoalveolar lavage; 1nt and 7nt, 1 and 7 nucleotides different compared to the reference sequence; hsp65, heat-shock protein 65 gene

### Association between *T*. *whipplei* colonization, pulmonary dysfunction, and inflammation

**Fig 2:**
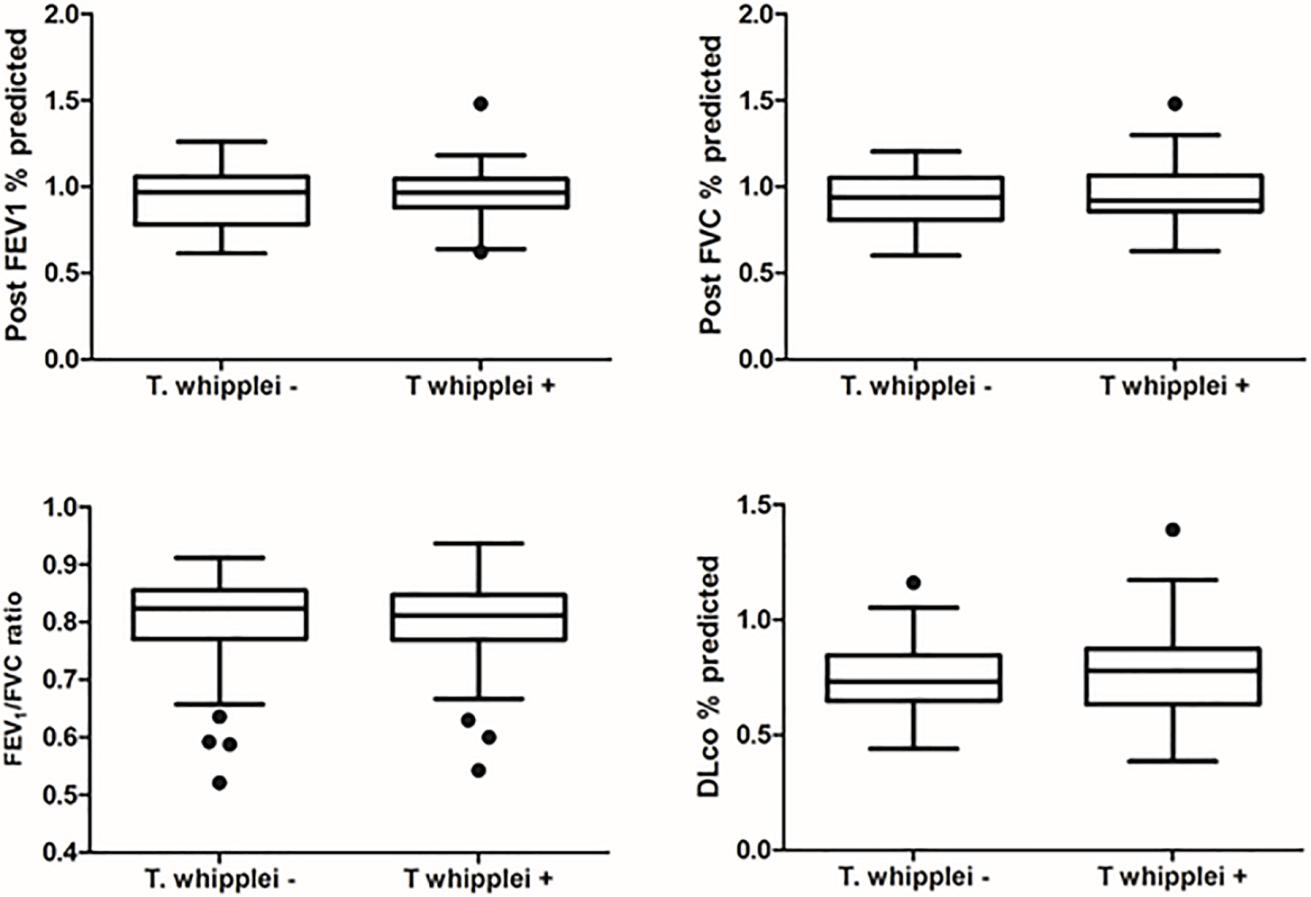
Measures of pulmonary function in HIV-infected individuals by *T*. *whipplei* status. Post-bronchodilator spirometry and diffusing capacity for DLco were performed for each participant per American Thoracic Society guidelines. Measures of pulmonary function were analyzed by *T*. *whipplei* status using Kruskal-Wallis test for continuous variables and Fisher exact test for categorical. There were no significant differences in pulmonary function test parameters by *T*. *whipplei* status. FEV1, forced expiratory volume in one second; FVC, forced vital capacity; DLco, diffusing capacity for carbon monoxide.

**Fig 3:**
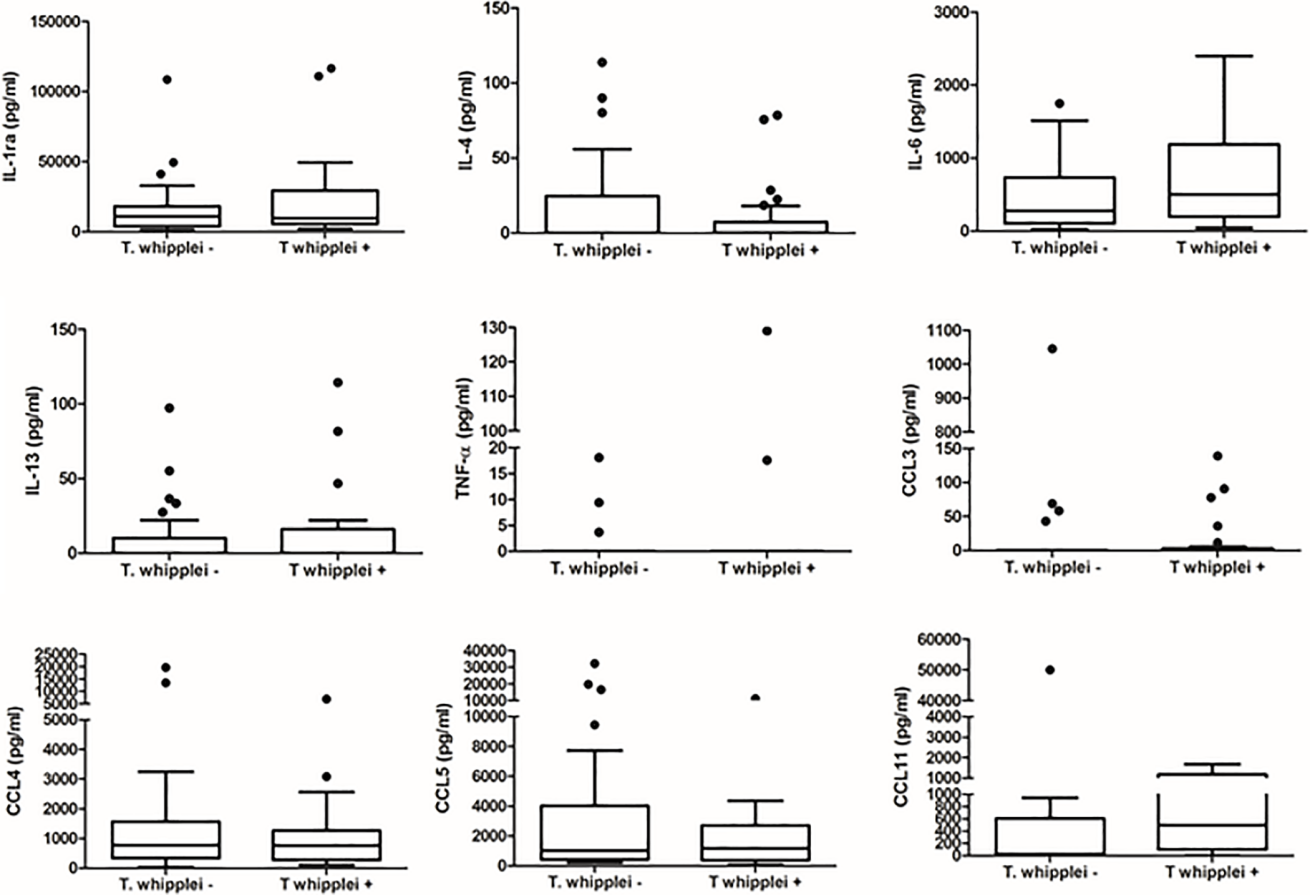
Levels of proinflammatory biomarkers in bronchoalveolar lavage by *T*. *whipplei* status. Levels of proinflammatory cytokines and chemokines in BAL supernatant were measured using a Luminex assay kit from Bio-Rad per the manufacturer’s protocol. Data were analyzed by *T*. *whipplei* status using Kruskal-Wallis test for continuous variables and Fisher exact test for categorical. There were no significant differences in levels of cytokine and chemokine by *T*. *whipplei* status.IL-1RA, interleukin-1 receptor antagonist.

**Fig 4:**
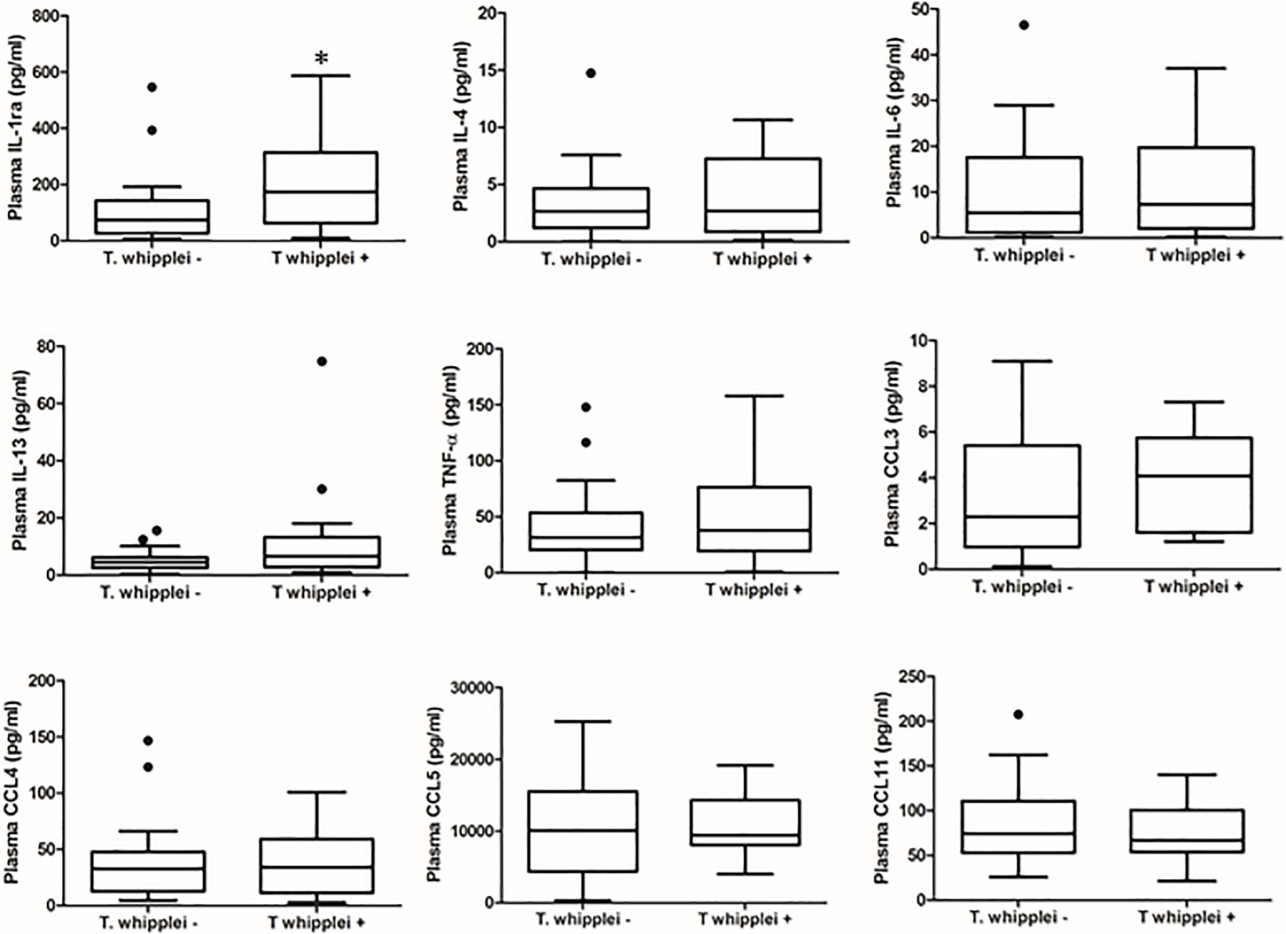
Levels of proinflammatory biomarkers in plasma by *T*. *whipplei* status. Levels of proinflammatory cytokines and chemokines in plasma were measured using a Luminex assay kit from Bio-Rad per the manufacturer’s protocol. Data were analyzed by *T*. *whipplei* status using Kruskal-Wallis test for continuous variables and Fisher exact test for categorical. There were no significant differences in levels of cytokine and chemokine except IL-1RA by *T*. *whipplei* status. IL-1RA, interleukin-1 receptor antagonist. *P = 0.025.

**Fig 5:**
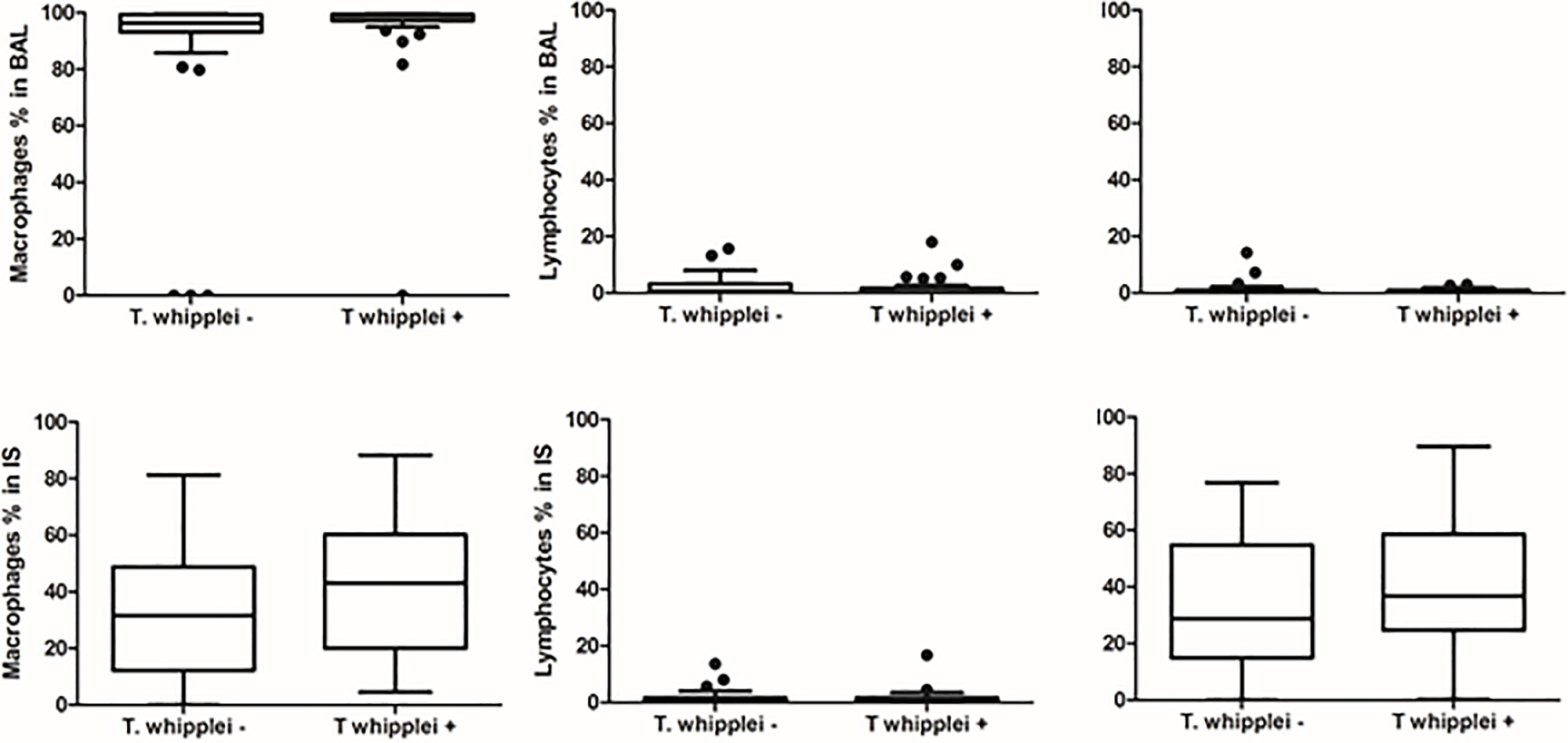
Cytology of bronchoalveolar lavage and induced sputum samples by *T*. *whipplei* status. Differential cell counts in BAL fluid and IS were performed to determine the numbers and percentages of inflammatory cells. Data were analyzed by *T*. *whipplei* status using Kruskal-Wallis test for continuous variables and Fisher exact test for categorical. There were no significant differences in percentages of different inflammatory cell types by *T*. *whipplei* status.

Demographic data, peripheral CD4^+^ cell count, plasma HIV RNA viral level, ART use and history of prior pneumonia did not differ significantly by *T*. *whipplei* status (Table 1). There was a trend for *T*. *whipplei-*colonized participants to be more likely to be female and smokers. Overall, 49 of 76 participants (64.5%) had at least one abnormality in pulmonary function tests (PFTs) with 12 of 76 (15.8%) having a post-bronchodilator forced expiratory volume in one second (FEV1)/forced vital capacity (FVC) <0.70 and 44 of 76 (57.9%) having a DLco percent predicted <80%. Analyses of pulmonary function parameters, specifically demonstrated that there were no significant differences in FEV1, FVC, FEV1/FVC and DLco between the *T*. *whipplei*-positive and negative groups based on either BAL or IS (Fig 2). Twenty-one of 49 (42.9%) participants with abnormal lung function had *T*. *whipplei* detected in the lungs, similar to the overall prevalence of *T*. *whipplei* (43.4%) in the cohort. After adjusting for effects of gender and smoking, there was no statistically significant difference between *T whipplei*-positive and negative participants forFEV1% (P = 0.61), FVC% (P = 0.37), FEV1/FVC (P = 0.63) and DLco% (P = 0.44) as well as FEV1/FVC below 70 percent (P = 0.40) and DLco below 80 percent predicted (P = 0.59). In a penalized logistic regression and after adjusting for smoking status and gender, the effect of *T*. *whipplei* on FEV1/FVC<0.7 (Odds ratio = 1.73, 95%CI = 0.49–6.13) and DLco<0.8 (Odds ratio = 0.76, 95%CI = 0.27–2.08) was not significant. We did not detect significant differences between *T*. *whipplei*-positive and negative participants in the levels of 9 proinflammatory biomarkers in BAL and plasma except plasma IL-1RA (Figs 3 and 4). In addition, we did not find significant differences in percentages of inflammatory cells in BAL fluid and IS samples by *T*. *whipplei* status (Fig 5).

## Discussion

This study investigated the concordance of BAL and IS detection of *T*. *whipplei* and the relationship between *T*. *whipplei* colonization in the lung, pulmonary dysfunction, and lung and systemic inflammation in HIV-infected individuals. We used *T*. *whipplei* hsp65 specific nested-PCR and sequencing approaches to identify and confirm *T*. *whipplei* in BAL and IS samples and found that 43.4% of HIV-infected individuals had *T*. *whipplei* colonization in the lungs. We found concordance between BAL and IS samples in the detection of *T*. *whipplei*. We did not find independent associations between pulmonary *T*. *whipplei* colonization and lung and systemic inflammation or pulmonary dysfunction.

The *T*. *whipplei* hsp65 specific-PCR assay is based on the *T*. *whipplei* hsp65 gene sequence and has very high sensitivity and specificity [24]. Using this assay, Morgebegg and colleagues detected *T*. *whipplei* in gastric aspirates, intestinal tissue and saliva samples from ten individuals without active Whipple’s disease [24]. In this study, we detected a higher frequency (43.4%) of *T*. *whipplei* colonization in the lungs of HIV-infected individuals compared to the 18.5% frequency in healthy individuals reported previously using PCR [27]. Using 16S rRNA gene sequencing, frequency of *T*. *whipplei* in BAL was previously determined to be about 13.0% in healthy individuals and 31.7% in HIV-infected individuals [4]. In bronchial brush samples, the frequency was 23.4% in HIV-infected individuals [28]. In 39 BAL samples from this study, 33.3% (13/39) and 30.8% (12/39) were detected *T*. *whipplei* by *T*. *whipplei* PCR and 16s rRNA gene sequencing, respectively, indicating that *T*. *whipplei* hsp65 specific-PCR assay has greater sensitivity than 16S rRNA gene sequencing. Simpson reported that the *T*. *whipplei* PCR assay was more sensitive than 16S rRNA sequencing for *T*. *whipplei* detection in IS of individuals with asthma [29]. These results indicate that *T*. *whipplei* hsp65 specific-PCR assay can be used for detection of *T*. *whipplei* colonization in lung.

Relative characteristics of BAL versus IS for detection of *T*. *whipplei* are unknown, but sensitivity and specificity were high for IS when compared to BAL. In addition, there was strong genetic identity of *T*. *whipplei* detected in the BAL and IS samples from the same participants, and individuals with a positive BAL were more likely to have positive IS. These results indicate that IS may also be useful to detect *T*. *whipplei* colonization in the lung and may detect some cases not sequenced in BAL as it samples a wider area of alveoli.

HIV-infected individuals have a high prevalence of pulmonary function abnormalities. In our cohort, 15.8% of participants had a post-bronchodilator FEV1/FVC less than 70 percent, 57.9% had a DLco percent predicted less than 80 percent predicted, and 64.5% had at least one pulmonary abnormality. Detection of certain organisms, such as *P*. *jirovecii*, at low levels in the lungs of HIV-infected individuals has been associated with pulmonary inflammation and COPD [13,14]. Whether *T*. *whipplei* in the lungs of HIV-infected individuals increases local and systemic inflammation and worsens pulmonary function has not previously been investigated. In this study, we did not find significant associations between *T*. *whipplei* colonization, lung and systemic inflammation and pulmonary dysfunction in HIV-infected individuals. These results add to prior work conducted in HIV-uninfected individuals and animal studies[12,27]. We previously showed that *T*. *whipplei* was detectable in the lung in HIV-uninfected healthy individuals, but there was not a significant association between presence of *T*. *whipplei* and decreased pulmonary function [27]. Sze and colleagues have detected *T*. *whipplei* in bronchial brush samples in HIV-infected individuals, but did not find any difference in microbial diversity and richness between HIV-infected individuals with and without COPD [28]. In a SHIV-infected macaque model, we also did not find a relationship between *T*. *whipplei* colonization and the development of COPD [12].

There are several limitations to our study. First, we may have lacked power to see some associations. The current study has 80% power to detect a difference of 11% or more between *T*. *whipplei* groups for post FVC%. A total sample size of 540 (including 310 *T*. *whipplei*—and 230 *T*. *whipplei* +) is required to gain 80% power for detecting the current difference of 4% significantly. Our previous work in determining the impact of *Pneumocystis* colonization in HIV-infected individuals showed a statistically significant effect in 32 individuals[11]. The current study is more than twice as large as this previous cohort and still fails to find an association with *T*. *whipplei*. In addition, we do not see any trend towards differences in pulmonary function with colonization. There is not a strict definition of a large bronchoscopy study although given the difficulties and expense associated with research bronchoscopy, 76 participants are bigger than many prior studies [11,27]. In addition, selection of different inflammatory markers might have shown a relationship to *T*. *whipplei*. However, we chose these inflammatory markers because studies have shown that plasma levels of these cytokines are negatively associated with lung function in HIV-infected individuals [16,17]. In this cohort, 93.4% participants received ART, median CD4^+^ cell count was more than 600 cells/μl, and median plasma HIV viral load was about 100 copies per ml. Thus, we do not know if these results are consistent with those from the individuals with severe immune suppression. Finally, we only investigated presence or absence of *T*. *whipplei*. Gene activity of the organism, abundance of *T*. *whipplei*, or interaction of *T*. *whipplei* with other members of the microbial community may have more impact on lung inflammation and function.

Although we detected *T*. *whipplei* in the lungs of 43.4% participants of an HIV-infected cohort, there was no relationship of *T*. *whipplei* to either pulmonary and systemic inflammation or pulmonary function. Thus, there does not seem to be a basis to suggest that prevention or treatment of *T*. *whipplei* would impact lung function in HIV-infected individuals. However, future larger longitudinal studies with serial BAL, serial measurement of biomarkers and pulmonary function tests are needed.
